# Interplay between sleep and exercise in older adults at increased risk of Alzheimer disease and related dementias

**DOI:** 10.1002/alz70860_098142

**Published:** 2025-12-23

**Authors:** Connor Snow, Sophie Berghmans, Quinn Smith, Alison Donald, Matiram Pun, Shane Magnison‐Benoit, Jean M Rawling, Jessalyn K Holodinsky, Stewart Longman, Marc J Poulin

**Affiliations:** ^1^ University of Calgary, Cumming School of Medicine, Calgary, AB, Canada; ^2^ University of Calgary, Faculty of Arts, Calgary, AB, Canada

## Abstract

**Background:**

Alzheimer disease (AD) and related dementias (ADRD) are a global concern, with major personal and socio‐economic impacts. Physical inactivity and poor sleep are important modifiable risk factors for ADRD and addressing these factors could reduce the prevalence of dementia by up to 40%. For example, poor sleepers have a 1.6‐fold higher risk of developing AD but how do we define poor sleep, what components of sleep are relevant, by how much can sleep quality be improved and what effect does improving sleep have on brain health? Equally, what types of physical activity improve brain health and sleep quality, and what are the relative magnitudes of effect? We will present data demonstrating the association between poor sleep and impaired cognition, and the beneficial effects of aerobic exercise on sleep and cognition in older adults at increased risk of ADRD.

**Methods:**

Sleep (in‐home polysomnography), cardiorespiratory fitness (VO_2_peak) and cognition (overall composite, domains) data from two cohorts: BIM‐I is a prospective cohort study of older sedentary adults with longitudinal assessments over a long term (5‐7 years) follow‐up. The second cohort is from the BIM‐II randomized controlled trial involving aerobic exercise in older adults at increased risk of ADRD. Data from BIM‐II examines the inter‐relationship between exercise, sleep and cognition.

**Results:**

Data from BIM‐I and II demonstrate: 1) compared to younger adults, older adults have a significant reduction in deep (i.e., slow wave) sleep, an increase in transitional sleep and more wakefulness during sleep; 2) evidence of sex differences in sleep and in exercise effects on sleep; and 3) aerobic exercise is more effective than stretch and tone exercise to improve sleep quality, cognition, and cerebrovascular health.

**Conclusions:**

The novel cross‐sectional, interventional and longitudinal data emerging from BIM‐I and BIM‐II in community dwelling & non‐clinical populations provide evidence supporting age‐related declines in sleep architecture and improvements in sleep with aerobic exercise, as well as a complex interplay between sleep, exercise and cognition in older adults at increased risk of ADRD. These findings help advance knowledge of how lifestyle modifications maintain brain and cognitive health with aging, with relevance to prevention of ADRD.

